# Duplicated genes find their space: Spatial transcriptomics illuminates evolutionary fates

**DOI:** 10.1093/plcell/koaf265

**Published:** 2025-11-01

**Authors:** Min-Yao Jhu, Fabian van Beveren, Bruno Guillotin

**Affiliations:** Assistant Features Editor, The Plant Cell, American Society of Plant Biologists; Crop Science Centre, Department of Plant Sciences, University of Cambridge, Cambridge CB3 0LK, UK; Laboratory of Microbiology, Wageningen University & Research, Wageningen 6708 WE, The Netherlands; Institute of Plant Sciences Paris-Saclay (IPS2), CNRS, INRA, Univ d’Evry, Université de Paris; Université Paris-Saclay, Gif-sur-Yvette 91190, France

Gene and genome duplications are fundamental forces driving plant evolution, leading to relaxed selection and expanding genetic toolkits (Van de Peer et al. 2017; Almeida-Silva and Van de Peer 2023). However, the subsequent fate of these duplicates—whether they retain redundant functions, divide tasks (subfunctionalization), or acquire new roles (neofunctionalization)—largely depends on how they originated (Birchler and Yang 2022). While previous evolutionary studies relied primarily on bulk RNA sequencing, which aggregates expression profiles across entire tissues, recent research published by Almeida-Silva and Van de Peer uses spatial transcriptomics across 5 diverse plant species (*Arabidopsis thaliana*, soybean, orchid, maize, and barley) to reveal the evolution of gene expression divergence at the cell-type level (Figure A). This provides a spatially resolved view of how the mode of duplication impacts the expression of duplicated genes across angiosperms.

**Figure.**
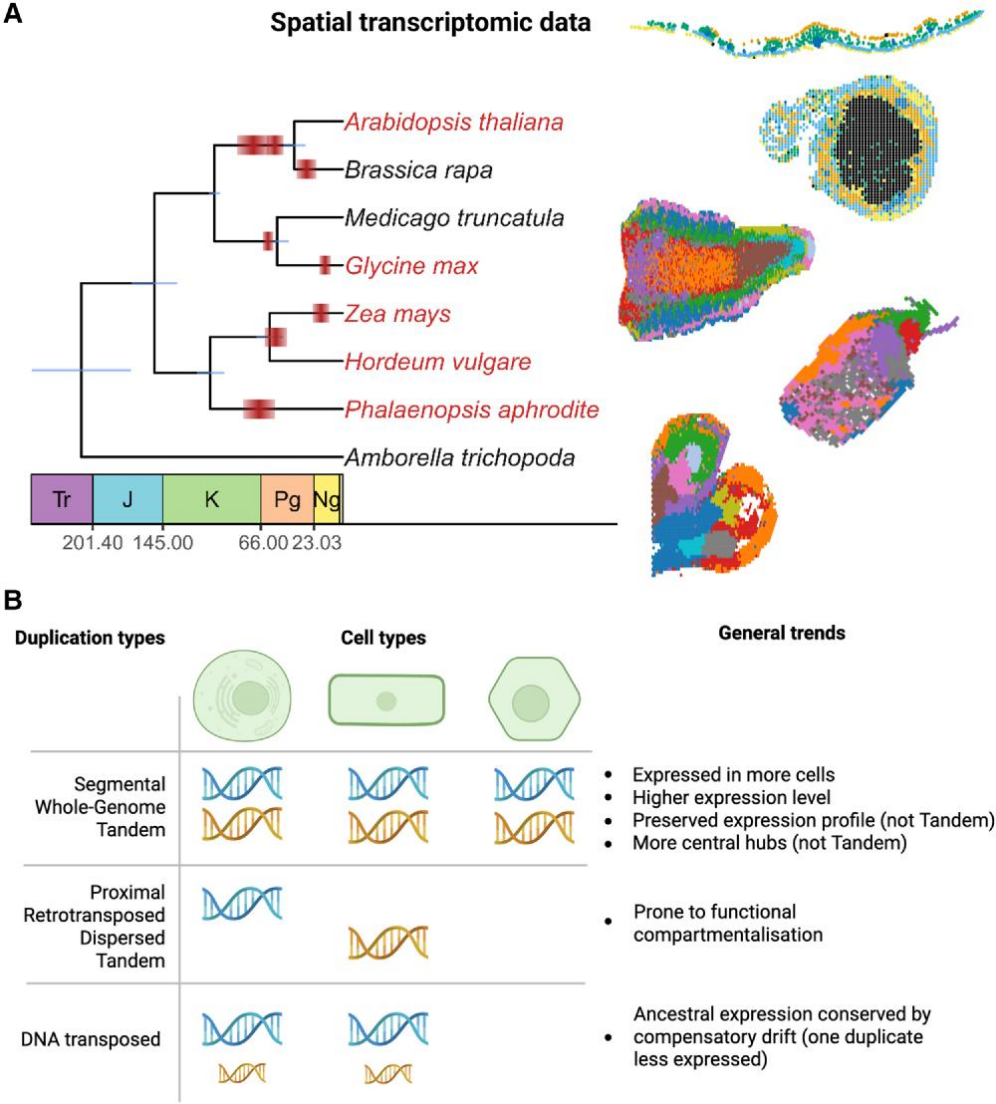
Spatial transcriptomics reveals how duplication mode influences gene fate. **A)** Phylogenetic relationships among the 5 analyzed species, with rectangles indicating WGD events based on AngioWGD (Chen et al. 2025). Species included in the study are highlighted in red. Geological time abbreviations: Tr, Triassic; J, Jurassic; K, Cretaceous; Pg, Paleogene; Ng, Neogene. Numbers below denote boundary ages in millions of years ago. Representative spatial transcriptomic datasets are shown from top to bottom: *Arabidopsis thaliana* (Ath, leaf), soybean (*Glycine max*, Gma, nodule), orchid (*Phalaenopsis aphrodite*, Pap, inflorescence), maize (*Zea mays*, Zma, developing ear), and barley (*Hordeum vulgare*, Hvu, germinating seed). **B)** Conceptual summary of the fates of duplicated genes described in Almeida-Silva and Van de Peer (2025). While most duplicates exhibit asymmetric expression divergence, each duplication type tends to show distinct and specific evolutionary outcomes. This figure illustrates enriched duplicate-type behavior relative to other duplication modes; however, these general trends can vary among the plant species analyzed. Figure adapted from Almeida-Silva and Van de Peer (2025), Supplementary Fig. 1 and Fig. 2A.

The authors confirm a major paradigm: duplication mechanisms that preserve the cis-regulatory landscape generally yield paralogs with more preserved expression profiles (Figure B). Genes originating from segmental or whole-genome duplication (SD/WGD) and tandem duplication (TD) events often exhibit greater expression levels and are expressed in more cell types compared with genes derived from dispersed duplications. This preservation is hypothesized to be linked to the retention of ancestral transcription factor binding sites in promoters and enhancers. These large-scale duplicates are also found to be overrepresented in spatially variable gene sets, indicating variable expression across tissue compartments. Furthermore, SD/WGD-derived genes frequently hold central roles as hubs within coexpression networks, a finding consistent with the preferential retention of essential, dosage-sensitive genes following SD/WGD events (Jeong et al. 2001).

When classifying the eventual expression outcome of these duplicates, a clear separation emerges based on the scale of the duplication. Paralogs from large-scale duplications (SD/WGD) and small-scale DNA-transposed duplications (dTRD) typically display redundant or overlapping expression profiles across cell types more frequently than expected, suggesting functional redundancy or subfunctionalization in certain species. Conversely, other small-scale duplicates, including TD, proximal duplications, retrotransposed duplications, and dispersed duplications, often diverge asymmetrically. One gene maintains broad expression while the other specializes in a few cell types, a hallmark of functional compartmentalization (Figure B).

Crucially, the high-resolution data unveiled a fascinating mechanistic exception within the small-scale group: dTRD. Although dTRD genes do not preserve cis-regulatory elements to the same extent as SD/WGD or TD genes, they are nonetheless often overrepresented in pairs displaying expression-level redundancy (Figure B). The researchers determined that dTRD redundancy is not due to regulatory preservation but rather is better explained by “compensatory drift”: one copy evolves toward lower expression while the paralog evolves toward higher expression, thereby maintaining the overall total ancestral expression level (Iohannes and Jackson 2023). This leads to dTRD pairs displaying significantly greater absolute differences in expression levels between paralogs across cell types compared with other modes of duplication.

The study also underscores that the impact of the duplication mode is not static: time is a crucial factor. While young duplicates (less than 20 million years ago) clearly show lower divergence when resulting from mechanisms that preserve regulatory landscapes (SD, TD, proximal duplications), these differences based on duplication mode disappear or fade over time in more ancient duplicates.

Finally, gene function imposes strong constraints on expression divergence. Dosage-sensitive genes involved in basic cellular processes (like chromatin assembly or transcription regulation) display highly preserved expression profiles, likely due to selection pressures maintaining stoichiometric balance (Birchler and Veitia 2012). Meanwhile, genes involved in specialized processes, such as flowering and phytohormone biosynthesis, diverge much more rapidly.

Collectively, by leveraging spatial transcriptomics, this research not only confirms that established theories of gene expression evolution following duplication hold true within tissue compartments but also provides mechanistic insights, like compensatory drift, which explain divergence outcomes when traditional cis-regulatory preservation is lacking. By mapping expression divergence within tissues and across cell types, they reveal how the combination of duplication mode, gene function, and evolutionary time impacts the transcriptional fate of gene duplicates and by extension shapes plant genome evolution and cell identity. While these overarching evolutionary outcomes and patterns are broadly conserved, the degree of divergence and functional specialization can vary among species, as also shown in this work, highlighting a potential future direction to uncover lineage-specific evolutionary trajectories.

## Recent related articles in *The Plant Cell*:

- Birchler and Yang (2022) discussed the multiple evolutionary fates of duplicated genes—including deletion, subfunctionalization, neofunctionalization, dosage balance, and neutral variation—highlighting how duplication mechanisms and functional constraints shape plant genome evolution.
- Van de Peer et al. (2021) reviewed how whole-genome duplications and polyploidy contribute to plant survival and diversification, emphasizing their role in adaptation to abiotic and biotic stress.
- Li et al. (2016) showed that core gene families display consistent duplicability across angiosperms, with single-copy genes linked to genome stability and multicopy genes to signaling and metabolism.

## Dive Curated Terms

The following phenotypic, genotypic, and functional terms are of significance to the work described in this paper:
